# Supplementary material for: Patterns of Intron Gain and Loss in Fungi
Source: PLoS Biol. 2004 Nov 30;2(12):e422. doi: 10.1371/journal.pbio.0020422 (PMC532390; doi:10.1371/journal.pbio.0020422)
Supplement: Table S1 — Also available at http://genes.mit.edu/NielsenEtAl/. (4.3 MB ZIP). [file pbio.0020422.st001.zip › NielsenEtAl/html/940.html]

AN5193.1.NCU02543.1.MG01564.1.FG01389.1


```
 CLUSTAL W (1.82) Multiple Sequence Alignments - Introns Inserted


Sequence 1: AN5193.1	409 aa
Sequence 2: FG01389.1	398 aa
Sequence 3: MG01564.1	400 aa
Sequence 4: NCU02543.1	401 aa
Alignment Length: 434 aa
Number Identitical Residues: 145 aa
Alignment Score (without introns) 8009


MG01564.1 	MVRIEPFHVEQ~WMDKYETKQNVLNVSETCVSSQSIDDLLELSNDKSSICSVFS-TSRKL
NCU02543.1	MVKITPFEVEQ~WMDRYETTPGVINIAETCCASISIDDLISLNTDKSACSPLQ--LSKKL
FG01389.1 	MVRIAPFAVEQ~WMDKYETTAGVLNVAETCAASVSIDDLVGMCRDTQTPGPID--TSIKL
AN5193.1  	MVQIKDFAVEQ0WMDKYETTA-KYNVAETCSASISVRDLQALCEDPALN-PLDGVLDRKL
          	**:*  * *** ***:***.    *::*** :* *: **  :  *     .: .  . **

MG01564.1 	TYGAILGSESLRSSVAGLYS------TEAGTRLSPENVLITPGAIFANFLLYYT~LIGPG
NCU02543.1	TYGDILGSEILRQRVASLLNRDLNHHSPGLEPLNAENVIITQGAIAANFLTFYT~LVGPG
FG01389.1 	TYGAIPGSQVLRERIAARCS------SEG-TQLAAEDIIVTQGAIGANFLALYS2LIGPG
AN5193.1  	TYGEIRGSKQLRSTLANLYS------VRTPTPLPSDNILVTAGAIQANFLLLYT~LVGPG
          	*** * **: **. :*   .            * .:::::* *** ****  *: *:***

MG01564.1 	DHIVCVYPTYQQLYSVPQSLGAEVSLWRLSKENSYVPNMEELTGLVKTNTK0MIVVN~NP
NCU02543.1	DHVISVYPTYQQLYSVPESLGAEVSLWKLKAENGYVPDVDELEGLVKANTK0LLVLN~NP
FG01389.1 	DHVICVYPTYQQLYDVPRSIGAEVTLWKLKEEENFVPNTNDLVGMIQDNTK0MIIIN~NP
AN5193.1  	DHVICHYPTYQQLYSVPESLGAEVSLWKSKEAEGWKLDLEELKGLIRPNTK~LIIIN2NP
          	**::. ********.**.*:****:**: .  :.:  : ::* *::: *** ::::* **

MG01564.1 	NNPTGAPIPRGTLEEIVQFARRRNIIVFSDEVYRPLFHSLQ-KHVDQ-----PPSILSMN
NCU02543.1	NNPTGATIPTPTLQSILDFCSARNITILSDEVYSPLFHSLPASSSSATTSTAPPSILSLS
FG01389.1 	NNPTGVPIPDSVLERIAQIAKEKDVILFSDEVYRPLFHGGASGRMQA-----PQPATAFG
AN5193.1  	QNPTGAIIPQGTLDEIVEIARSSSIYVFCDEVYRPLFHSISPMDPDF-----PSSVLSLG
          	:****. **  .*: * ::.   .: ::.**** ****.  .   .      * .  ::.

MG01564.1 	------YDKAIATGSMSKAWSLAGVRVGWVACRDRSIIETMATARDYTTISVSQLDDQLA
NCU02543.1	PSGTAKTCVTLSTGSTSKALALAGLRLGWIVSRSPSLLRAIASARDYTAISVSQLDDQVA
FG01389.1 	------HEKSIVTGSMSKGYALAGIRVGWIASKDKSIISTIMAARDYTTISVSQIDDQIA
AN5193.1  	------YERAIVTGSLSKAYSLAGIRVGWIASRDRTVIEACASSRDYTTISVSQLDDAVA
          	         :: *** **. :***:*:**:..:. ::: :  ::****:*****:** :*

MG01564.1 	SFALSDPVRPALLDRNMKLAHRNLCLLEDFVKT-HDKICSWVKPQAGTTAFIQLS---KQ
NCU02543.1	SYALSPSVLPALLERNLDLARTNLALLTEFVEGKYSKVCSWVRPTAGTTALVQFRNLSKG
FG01389.1 	RYALSPAVLPSLVDRNLALARTNSRLIKEFIEG-HKSVCSWVEPTAGTTAFVRFNN--KN
AN5193.1  	SYALAPTTIHALLKRNIELGRTNLGILEKFIES-HRWACDWVKPRAGTTAFVRFN---KM
          	 :**: ..  :*:.**: *.: *  :: .*::  :   *.**.* *****::::    * 

MG01564.1 	GKPVDDEKFCIDAINTINVMLVPGSKCFGNGVDGDFKG~YIRLGYACETDVLKEALKRLG
NCU02543.1	GVPVDDATFVLDVLDKTHVLFMPGSPCFGLGTE--FKG2LAKLMFLEASQ----------
FG01389.1 	GEPVNDVDFCLDLLHKNNVLLVAGSKCFGNDKD--FKG~YIRMGYVCETDVLVEGLKRLG
AN5193.1  	GKPVNDTAFCEMLLERTGVMLVPGSLCFGGGED--FLG~YVRIGYVCETQVLEEGLAKLK
          	* **:*  *    :.   *:::.** *** . :  * *   :: :   ::   ..     

MG01564.1 	GYIEQNLS-----------
NCU02543.1	-------------------
FG01389.1 	TFIESNLL-----------
AN5193.1  	AFLEDDYEEVPAVKRKTQS
          	   ...  . .: . .:.:
```
